# Supplementary material for: Natural history of eukaryotic DNA viruses with double jelly-roll major capsid proteins
Source: bioRxiv. 2024 Mar 18:2024.03.18.585575. Preprint. [Version 1] doi: 10.1101/2024.03.18.585575 (PMC11071308; doi:10.1101/2024.03.18.585575)
Supplement: Supplement 1 [file NIHPP2024.03.18.585575v1-supplement-1.pdf]

bioRxiv preprint doi: https://doi.org/10.1101/2024.03.18.585575; this version posted March 18, 2024. The copyright holder for this preprint (which was not certified by peer review) is the author/funder. This article is a US Government work. It is not subject to copyright under 17 USC 105 and is also made available for use under a CC0 license.

SI figures and legends

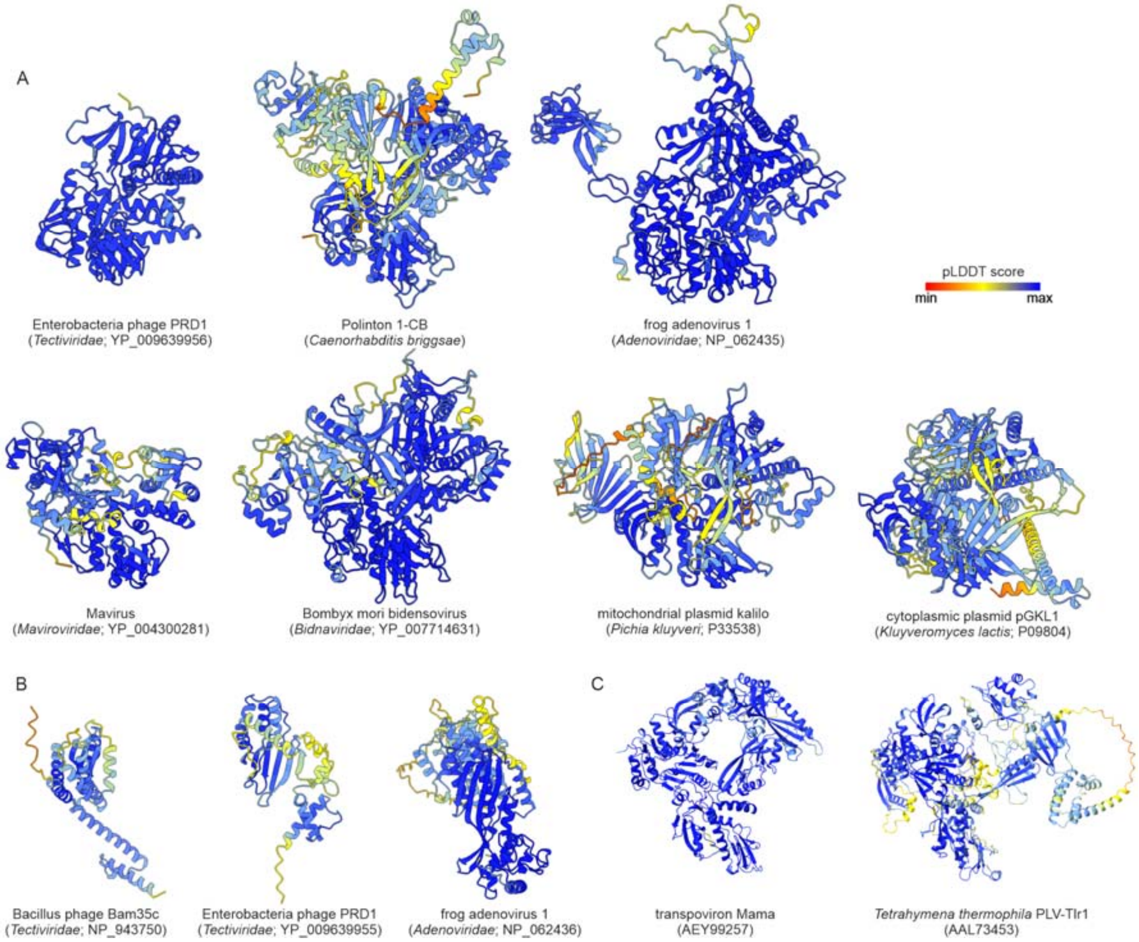

Figure S1. Structural models colored according to their per-residue confidence scores assessed by predicted local distance difference test (pLDDT). The pLDDT scale is show in in the top right corner of the figure. GenBank or UniProt accession numbers of the modeled proteins are provided in parentheses. A. Protein-primed family B DNAPs. B. Terminal proteins. C. Tlr1-like helicases.

bioRxiv preprint doi: https://doi.org/10.1101/2024.03.18.585575; this version posted March 18, 2024. The copyright holder for this preprint (which was not certified by peer review) is the author/funder. This article is a US Government work. It is not subject to copyright under 17 USC 105 and is also made available for use under a CC0 license.

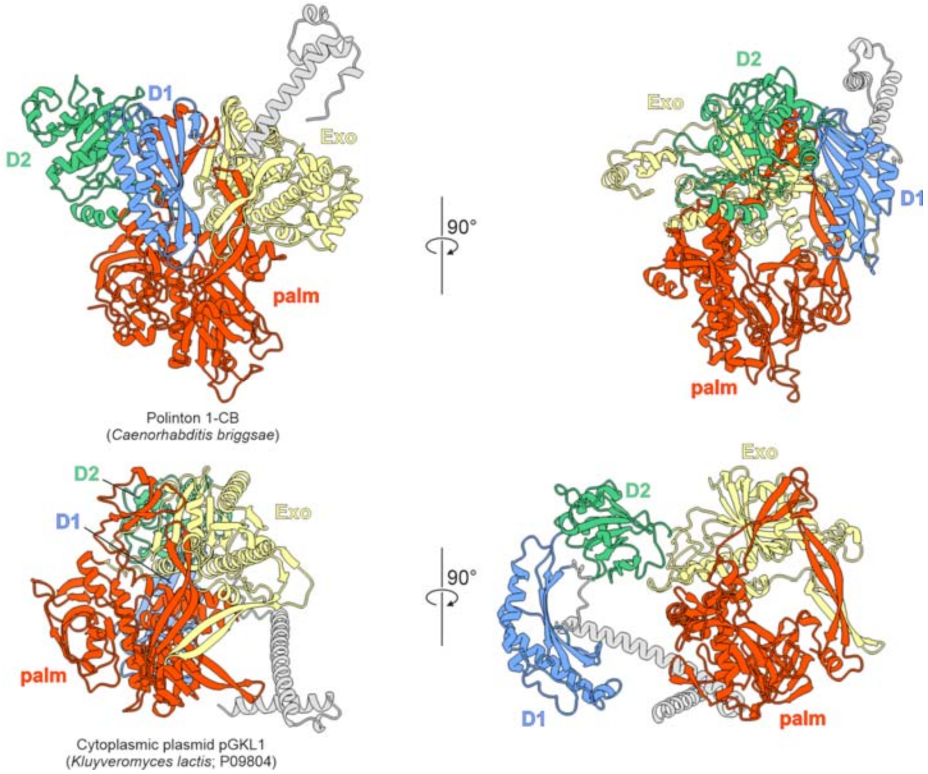

Figure S2. Comparison between structural models of the pPolBs encoded by polinton 1-CB and cytoplasmic linear plasmid pGKL1 from a yeast (*Kluyveromyces lactis*). The view on the left is in the same orientation as in Figure 2B, whereas the view on the right is rotated by 90°, so that D1 and D2 of pGKL1 pPolB become visible. Domains are colored as in Figure 2B.

bioRxiv preprint doi: https://doi.org/10.1101/2024.03.18.585575; this version posted March 18, 2024. The copyright holder for this preprint (which was not certified by peer review) is the author/funder. This article is a US Government work. It is not subject to copyright under 17 USC 105 and is also made available for use under a CC0 license.

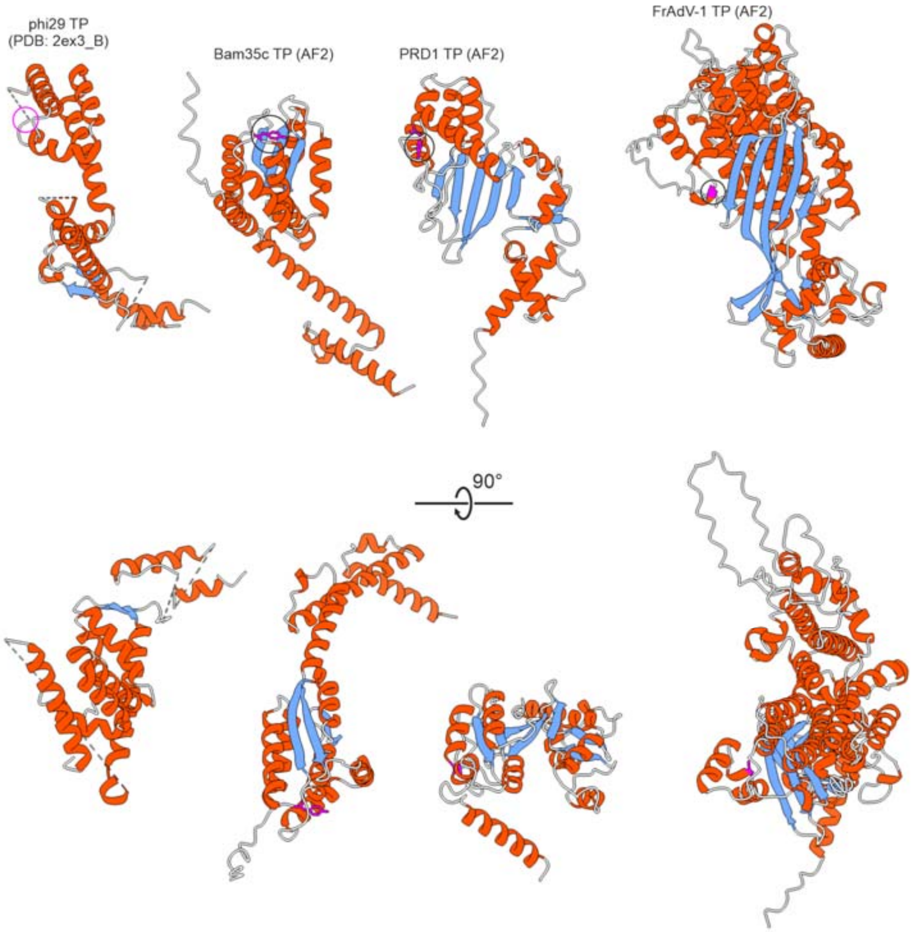

Figure S3. Structural comparison of terminal proteins. Comparison of the X-ray structure of the terminal protein (TP) of Bacillus subtilis phage phi29 with the structural models of terminal proteins encoded by tectivirids Bacillus phage Bam35c and Enterobacteria phage PRD1, and frog adenovirus 1. The structures are colored according to secondary structure elements: α-helices, red; β-strands, blue; coils, grey. The linking residues are circled. Note that the linking residue has not been resolved in the Bacillus subtilis phage phi29 structure (PDB: 2ex3_B).

bioRxiv preprint doi: https://doi.org/10.1101/2024.03.18.585575; this version posted March 18, 2024. The copyright holder for this preprint (which was not certified by peer review) is the author/funder. This article is a US Government work. It is not subject to copyright under 17 USC 105 and is also made available for use under a CC0 license.

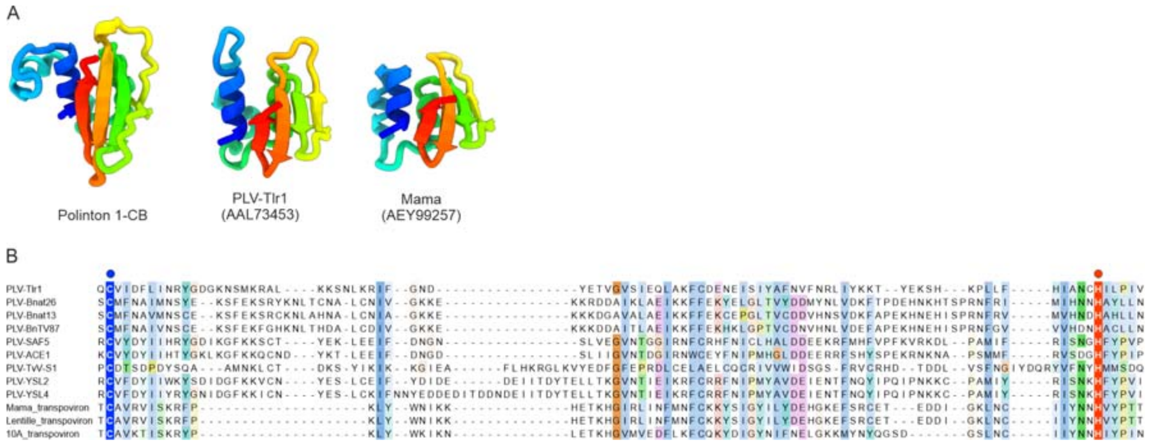

Figure S4. vOTU domains of Tlr1-like helicases. A. Comparison of the vOTU structural models from the pPolB of polinton 1-CB with the corresponding domains from the Tlr1-like helicases of PLV-Tlr1 and transpoviron Mama. The models are colored using the rainbow scheme from N-terminus (blue) to C-terminus (red). B. Sequence alignment of the vOTU domains of Tlr1-like helicases encoded by PLVs and transpovirons. The catalytic Cys and His residues are indicated with blue and red circles, respectively.

bioRxiv preprint doi: https://doi.org/10.1101/2024.03.18.585575; this version posted March 18, 2024. The copyright holder for this preprint (which was not certified by peer review) is the author/funder. This article is a US Government work. It is not subject to copyright under 17 USC 105 and is also made available for use under a CC0 license.

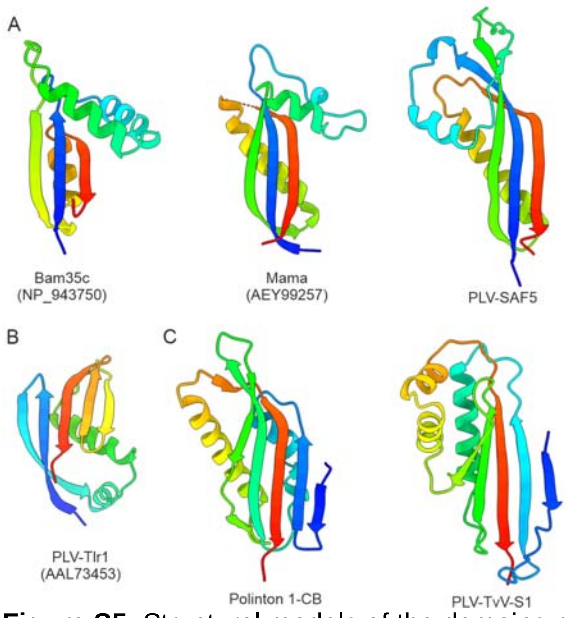

Figure S5. Structural models of the domains preceding the vOTU domain in Tlr1-like helicases and their comparison to the terminal proteins. Three structurally distinct domains are shown. A. Domain showing similarity to the terminal protein of tectivirid Bacillus phage Bam35c. B. Tlr1 element-specific domain. C. The domain of PLV TvV-S1 is compared to the PRD1-like terminal protein domain of polinton 1-CB. The models are colored using the rainbow scheme from N-terminus (blue) to C-terminus (red). PLV, polinton-like virus; TvV-S1, Tetraselmis viridis virus S1,

bioRxiv preprint doi: https://doi.org/10.1101/2024.03.18.585575; this version posted March 18, 2024. The copyright holder for this preprint (which was not certified by peer review) is the author/funder. This article is a US Government work. It is not subject to copyright under 17 USC 105 and is also made available for use under a CC0 license.

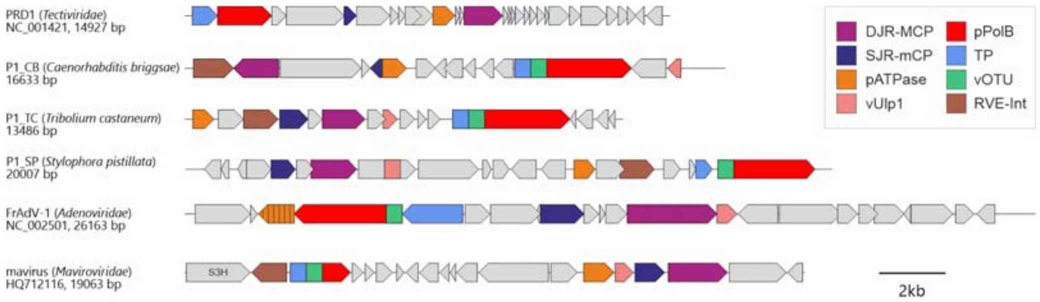

Figure S6. Genome maps of selected preplasmiviricots. Relevant genes are color-coded.

bioRxiv preprint doi: https://doi.org/10.1101/2024.03.18.585575; this version posted March 18, 2024. The copyright holder for this preprint (which was not certified by peer review) is the author/funder. This article is a US Government work. It is not subject to copyright under 17 USC 105 and is also made available for use under a CC0 license.

SI table

SI Table S1. Results of the DALI searches queried with the structural models of the terminal protein (TP) and vOTU domains

Domain	Element	Hit chain	Z score	RMSD, Å	lali, aa	nres, aa	%id	DALI hit
TP/D1	*Caenorhabditis briggsae* polinton 1 (P1-CB)	3oha-A	8.5	2.9	101	516	12	Yeast DNA polymerase eta (Y family)
		1t3n-A	7.9	4	103	388	7	Human DNA polymerase Iota (Y family)
		4ir1-A	7.8	3	91	342	4	Escherichia coli DNA polymerase IV (Y family)
	Mavirus	4ir1-A	6.2	3.8	92	342	8	Escherichia coli DNA polymerase IV (Y family)
		3bq1-A	6.1	4.1	98	344	10	Sulfolobus acidocaldarius DinB polymerases Dbh (Y family)
		1unn-D	6	2.8	84	112	7	Escherichia coli DNA polymerase IV (Y family)
	Kluyveromyces lactis plasmid pGKL1	3oha-A	6.8	3.8	105	516	8	Yeast DNA polymerase eta (Y family)
		3bq1-A	6.3	3.4	89	344	11	Sulfolobus acidocaldarius DinB polymerases Dbh (Y family)
		4ecw-A	6.2	3.9	100	432	3	Human DNA polymerase eta (Y family)
	Kalilo plasmid	3oha-A	7.7	3.4	104	516	8	Yeast DNA polymerase eta (Y family)
		4f4z-A	7.5	2.7	97	342	9	Dpo4-Dbh chimeric polymerase (Y family)
		3bq1-A	7.5	2.6	96	344	9	Sulfolobus acidocaldarius DinB polymerases Dbh (Y family)
	Bidnavirid	3oha-A	6.8	4	94	516	9	Yeast DNA polymerase eta (Y family)
		4ecw-A	5.9	3.5	85	432	9	Human DNA polymerase eta (Y family)
		6cst-A	5.8	3.1	83	446	14	Human DNA polymerase kappa (Y family)
	FrAdV-1	6cst-A	6.3	5.5	112	446	9	Human DNA polymerase kappa (Y family)
		3oha-A	6.1	4.2	128	516	6	Yeast DNA polymerase eta (Y family)
		7yll-A	5.9	5.6	123	329	7	Caldanaerobacter subterraneus DNA polymerase TTEDbh (Y family)
	Enterobacteria phage PRD1	6cst-A	3.4	4.3	99	446	13	Human DNA polymerase kappa (Y family)
vOTU/D2	*Caenorhabditis briggsae* polinton 1 (P1-CB)	7y5m-A	8.9	2.8	95	155	17	vOTU domain of Tacheng tick virus 1
		6dwx-B	8.4	2.9	92	156	17	vOTU domain of Qalyub virus
		4bop-B	8.2	2.8	101	150	11	OTU family deubiquitinase from Homo sapiense
	Mavirus	6ks5-B	7.2	2.6	85	313	18	Legionella pneumophila OUT deubiquitinase Ceg23
		4boq-A	7	2.6	83	175	12	OTU family deubiquitinase from Homo sapiense
		3c0r-A	6.9	2.5	82	175	12	OTU deubiquitinase from Saccharomyces cerevisiae
	FrAdV-1	7y5m-A	5.1	3.3	89	155	7	vOTU domain of Tacheng tick virus 1
		6dwx-B	4.6	3.5	84	156	13	vOTU domain of Qalyub virus
		4bou-A	4.5	2.9	85	141	6	OTU family deubiquitinase from Homo sapiense
	Kluyveromyces lactis plasmid pGKL1	7y5m-A	4	3	61	155	13	vOTU domain of Tacheng tick virus 1
		6dwx-B	3.5	2.5	60	156	12	vOTU domain of Qalyub virus
		6w9r-B	3.3	3	69	163	12	OTU deubiquitinase from Wolbachia pipientis
	Bidnavirid	6ks5-B	7.1	2.7	88	313	10	Legionella pneumophila OUT deubiquitinase Ceg23
		3pfy-A	4.8	2.5	74	129	9	OTU family deubiquitinase from Homo sapiense
		6dwx-B	4.6	3	75	156	12	vOTU domain of Qalyub virus
	Tlr1 element	6ks5-B	5.9	2.9	91	313	8	Legionella pneumophila OUT deubiquitinase Ceg23
		7y5m-A	5.4	2.9	89	155	8	vOTU domain of Tacheng tick virus 1
		8c83-x	5.1	3.2	93	259	9	OTU deubiquitinase from Saccharomyces cerevisiae
	Transpoviron Mama	7y5m-A	6.5	2.6	77	155	8	vOTU domain of Tacheng tick virus 1
		6ks5-B	5.7	2.7	76	313	12	Legionella pneumophila OUT deubiquitinase Ceg23
		4a5u-A	5.5	2.9	76	148	11	vOTU domain of turnip yellow mosaic virus
